# Remarks on Hæmaturia Miasmatica

**Published:** 1887-12

**Authors:** A. N. Perkins

**Affiliations:** Sabine Pass, Texas


### REMARKS ON HÆMATURIA MIASMATICA AND ITS TREATMENT.

*By A. N. Perkins, M. D., Sabine Pass, Texas.*

For Daniel's Texas Medical Journal.

HÆMATURIA Miasmatica is perhaps the most serious and fatal disease incidental to a southern latitude. Unless treated early and properly, it is almost invariably fatal.

As it is considered by every one to be a disease of malarial origin, it is strange that it has so recently become familiar to the general practitioner of the south. Twenty years ago it was unknown, or, at least, a very rare disease, where now it prevails to an alarming extent.

Many old and experienced physicians who have spent years in malarial regions, and were perfectly familiar with intermittent and remittent fevers, neuralgia, and other diseases of a malarial origin, had never been called upon to treat a case of hæmaturia miasmatica until within the last few years.

I commenced the practice of medicine in a malarial country, and did an extensive practice for more than ten years before a case of this type came under my observation. This would seem to favor the opinion that malaria alone is not sufficient for the origination of the disease; that some other factor or influence is necessary to co-operate with malaria in order to develop this peculiar and dangerous type. What is this factor or influence, is an important inquiry?

I am inclined to the opinion that it is the improper and excessive use of quinine by the people, Of late years, quinine has been in the hands of every one, and has been used without stint or discrimination. It is considered a panacea for every evil. If a man is sick, he takes it to cure himself. If well, and in good health, he takes it to prevent getting sick. I may say, in some families it is almost an article of diet. Taken in this way, without any preparation of the system, the bowels constipated, the secretions locked up, the liver and spleen engorged, what is the effect on a person long suffering from malarial toxæmia? It paralyzes the vaso motor system of nerves, lowers arterial tension, and precipitates collapse and hemorrhage.

Some eminent physicians consider quinine to be the most important remedy during the paroxysm of the disease. Dr. Bartholow says, quinine in laige doses is the sheet anchor. I differ in opinion from Dr. Bartholow, and believe that quinine in large doses in the beginning of the disease, can not possibly do any good. In the first place, the stomach can not tolerate it, and even if retained, it would not be absorbed or taken up into the system. If given hypodermically it can only act as a paralyzer and depressor. In beginning the treatment, my first remedy is a hypodermic dose of morphine to quiet the patient, and relieve his distress. I immediately commence to give him calomel and antimony, until the secretory system is aroused, at the same time I use rubifacients and diuretics, such as Bitart Potas, Nitr Potas, to keep up the action of the kidney, and avoid one of the great dangers, which is hemorrhage in the stroma of the kidneys and uriniferous tubes, causing suppression of urine. I have no use for astringents. The danger is not from hemorrhage, but from suppression, caused by the formation of clots in the kidney and bladder. Keep the secretion flowing, and let it dissolve and carry the blood out of those organs. Spirits of turpentine ln diuretic doses with spirits nitre is sometimes of great service. Strychnine gr. 1-40, quinine grs. 1, should be administered every two hours to stimulate and sustain the nervous system. When the calomel begins to act, and not until then, do I increase the dose of quinine. The patient sometimes dies after the urine has cleared up and the symptoms have become favorable, for the want of proper support by nourishment and stimulants. I know of no remedy equal to strychnine to sustain the nervous system in a disease of so much nervous depression and prostration. When improvement begins, Mur. Tinct. Iron, Chlor. Potas. is a good combination for the kidneys, and strychnine and quinine as a tonic.

I have, in the above, briefly given my views and treatment of this disease, which is now so prevalent and fatal in some parts of our country.

